# Chemodiversity of Essential Oils From *Eugenia uniflora* L. Collected in Different Phytophysiognomies of the Coastal Region of Rio de Janeiro

**DOI:** 10.1002/cbdv.202501390

**Published:** 2025-07-17

**Authors:** Eduardo Barros Duarte Junior, Ygor Nunes Moreira, Camila da Silva Barbosa Pereira, Durval Reis Mariano Junior, Rosana Santos Cavalcante, Diego da Paixão Alves, Douglas Figueredo dos Reis Pinheiro, Kawany da Silva Burgiert, Igor Sampaio Fontes, Mariana Emerick Silva, Lidiane Barbosa Pedro, Andre Marques dos Santos, Pedro Corrêa Damasceno Junior, Marco Andre Alves de Souza

**Affiliations:** ^1^ Programa De Pós‐Graduação Em Química, Instituto de Química, Universidade Federal do Rio de Janeiro Rio de Janeiro Brazil; ^2^ Programa De Pós‐Graduação Em Ciencias Ambientais e Florestais, Instituto de Florestas Universidade Federal do Rio de Janeiro Rio de Janeiro Brazil; ^3^ Laboratório De Plantas Aromáticas e Medicinais Universidade Federal do Rio de Janeiro Rio de Janeiro Brazil; ^4^ Departamento De Bioquímica, Instituto de Química Universidade Federal do Rio de Janeiro Rio de Janeiro Brazil; ^5^ Departamento De Agrotecnologia e Sustentabilidade, Instituto de Agronomia Universidade Federal do Rio de Janeiro Rio de Janeiro Brazil

**Keywords:** GC–MS, Myrtaceae, pitanga, surinam cherry, volatiles oil

## Abstract

This study aimed to evaluate the yield, color, and chemical composition of *Eugenia uniflora* essential oils collected from different regions of the Rio de Janeiro (in situ), and to describe the observed diversity based on statistical analyses. A total of 42 samples were collected, essential oils were obtained by hydrodistillation of leaves, with yield expressed as percentage, and analyzed by GC–FID and GC–MS. Descriptive and multivariate statistical analyses were applied for chemical differentiation and classification. Essential oil yields ranged from 0.26% to 3.49%, with the lowest values observed in reddish samples. Seventy‐two compounds were detected, of which 49 were identified. Oxygenated sesquiterpenes were the most abundant constituents in the analyzed essential oils, accounting for an average of 69% of the natural product. Cluster analysis revealed three chemical profiles comprising the largest number of samples, a result corroborated by principal component analysis. Nine different chemotypes were identified, most of which were primarily composed of curzerene (**1**)—a cope rearrangement artifact—and selina‐1,3,7(11)‐trien‐8‐one (**7**). The yield, color, and chemical composition of *E. uniflora* essential oils varied among the samples analyzed. The identification of multiple chemotypes of *E. uniflora* highlights the chemical diversity and its potential applicability across various industrial sectors.

## Graphical abstract

Essential oils from Eugenia uniflora collected in different coastal phytophysiognomies of Rio de Janeiro showed notable chemodiversity. GC‐MS and GC‐FID analyses revealed nine chemotypes, mainly rich in curzerene and selina‐1,3,7(11)‐trien‐8‐one.

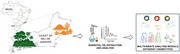

## 1 Introduction

Natural compounds have been extensively studied due to their therapeutic potential and the increasing interest in eco‐friendly substitutes for industrial sectors related to aromas and fragrances [1, 2]. Among these products, essential oils are characterized by a complex mixture of compounds, which may be influenced by environmental and genetic factors [3].

Scientific interest in characterizing the chemical diversity of essential oils has intensified, particularly in the context of valuing the genetic heritage of Brazilian biomes [4]. This valorization reflects not only the ecological and sociocultural importance of these resources but also their potential for sustainable economic exploitation. This perspective is especially relevant in the Atlantic Forest, one of the most biodiverse biomes on the planet, where native species such as *Eugenia uniflora* L. (Myrtaceae), commonly known as surinam cherry—or pitanga in Brazil—remain underutilized [5].

The Atlantic Forest is, unfortunately, one of the biomes most affected by urban expansion [6, 7, 8]. Unregulated growth and real estate development in ecologically sensitive areas, such as mangroves and *restingas*—coastal sandy plains with shrubby vegetation—have significantly contributed to the reduction of native vegetation cover, resulting in the genetic erosion of species such as *E. uniflora* [9]. This highlights the importance of conducting studies aimed at characterizing genetic diversity, collecting propagative material, and establishing ex situ collections in research institutions. Such actions are essential for conserving the genetic diversity and biological heritage of the Atlantic Forest and are also aligned with the Sustainable Development Goals (SDGs) proposed by the United Nations General Assembly [10, 11, 12].

*E. uniflora* predominantly occurs in *restinga* ecosystems, where it exhibits a shrubby growth habit, and in fragments of *floresta ombrófila densa*—dense evergreen rainforest typical of the Atlantic Forest biome—where it can develop into a small tree [13]. Its fruits are used for food, and its extracts and essential oils exhibit pharmacological properties, including hypoglycemic, antimicrobial, and antioxidant activities [14, 15]. The essential oil extracted from the leaves of *E. uniflora* is rich in sesquiterpenes such as curzerene, selina‐1,3,7(11)‐trien‐8‐one, and spathulenol, among others, which confer biotechnological potential to the species [16, 17, 18, 19].

Previous studies indicate that the essential oils of *E. uniflora* exhibit chemical diversity, possibly related to genetic and environmental factors. In natural populations from the state of Paraná (Brazil), for example, six different chemotypes were identified, demonstrating the intraspecific diversity of the species [18]. In addition, plant seasonality and phenological stage may influence the chemical composition of essential oils, as observed by other authors [20, 21, 22]. However, there is a knowledge gap regarding the chemical variability of *E. uniflora*, particularly in regions with distinct phytophysiognomies such as those found along the coast of Rio de Janeiro state.

This study revealed the chemical diversity of essential oils from *E. uniflora* collected along the coast of Rio de Janeiro. Hyphenated chromatographic techniques were employed to characterize the essential oils, and descriptive and multivariate statistical analyses were used to describe and organize the chemical profiles into major chemotypes.

## 2 Results

A total of 42 samples of *E. uniflora* were collected from different locations in the state of Rio de Janeiro (Table 1). The spatial distribution of the samples showed a higher concentration in the Norte Fluminense and Baixadas Litorâneas Regions (15 and 11 samples, respectively), followed by the Costa Verde and Metropolitana Regions (10 and 6 samples, respectively).

**TABLE 1:** Identification codes, herbarium voucher numbers, collection sites, and geographic coordinates of *Eugenia uniflora* plants collected in coastal regions of Rio de Janeiro, Brazil.

Plant ID	Herbarium ID	Municipality	Region	Longitude	Latitude
EU01	RBR 56110	Rio de Janeiro	Metropolitana	−43.603889	−22.997222
EU02	RBR 56109	Rio de Janeiro	Metropolitana	−43.538333	−23.049167
EU03	RBR 56108	Rio de Janeiro	Metropolitana	−43.521111	−23.047778
EU04	RBR 56107	Rio de Janeiro	Metropolitana	−43.493056	−23.033889
EU05	RBR 56106	Rio de Janeiro	Metropolitana	−43.444444	−22.932778
EU06	RBR 56105	Maricá	Metropolitana	−42.885000	−22.954722
EU07	RBR 56104	Maricá	Metropolitana	−42.800556	−22.959167
EU08	RBR 56103	Saquarema	Baixadas Litorâneas	−42.646111	−22.936389
EU09	RBR 56102	Saquarema	Baixadas Litorâneas	−42.458611	−22.933889
EU10	RBR 56101	Araruama	Baixadas Litorâneas	−42.313611	−22.936389
EU11	RBR 56100	Arraial do Cabo	Baixadas Litorâneas	−42.149444	−22.944444
EU12	RBR 56099	Arraial do Cabo	Baixadas Litorâneas	−42.050556	−22.956944
EU13	RBR 56098	Cabo Frio	Baixadas Litorâneas	−41.984722	−22.872222
EU14	RBR 56097	Armação dos Búzios	Baixadas Litorâneas	−41.954444	−22.813889
EU15	RBR 56096	Mangaratiba	Costa Verde	−43.990278	−22.937778
EU16	RBR 56095	Mangaratiba	Costa Verde	−44.151389	−23.035000
EU17	RBR 56094	Angra dos Reis	Costa Verde	−44.328611	−22.947778
EU18	RBR 56093	Angra dos Reis	Costa Verde	−44.419444	−22.947778
EU19	RBR 56092	Angra dos Reis	Costa Verde	−44.500556	−23.025000
EU20	RBR 56091	Paraty	Costa Verde	−44.556667	−23.036667
EU21	RBR 56090	Paraty	Costa Verde	−44.620833	−23.043333
EU22	RBR 56890	Paraty	Costa Verde	−44.678056	−23.063056
EU23	RBR 57578	Rio das Ostras	Baixadas Litorâneas	−41.932530	−22.527122
EU24	RBR 57579	Rio das Ostras	Baixadas Litorâneas	−41.931810	−22.534135
EU25	RBR 57580	Rio das Ostras	Baixadas Litorâneas	−41.914680	−22.508474
EU26	RBR 57581	Rio das Ostras	Baixadas Litorâneas	−41.897569	−22.491522
EU27	RBR 57582	Macaé	Norte Fluminense	−41.820042	−22.417502
EU28	RBR 57583	Macaé	Norte Fluminense	−41.686680	−22.297902
EU29	RBR 57584	Macaé	Norte Fluminense	−41.708305	−22.291248
EU30	RBR 57585	Campos dos Goytacazes	Norte Fluminense	−41.023452	−22.027123
EU31	RBR 57586	Campos dos Goytacazes	Norte Fluminense	−40.995348	−22.006428
EU32	RBR 57587	Campos dos Goytacazes	Norte Fluminense	−40.988973	−22.000455
EU33	RBR 57963	Campos dos Goytacazes	Norte Fluminense	−40.983298	−21.989155
EU34	RBR 57964	São João da Barra	Norte Fluminense	−41.003870	−21.928682
EU35	RBR 57965	São João da Barra	Norte Fluminense	−40.985070	−21.915020
EU36	RBR 57966	São João da Barra	Norte Fluminense	−41.026349	−21.736204
EU37	RBR 57967	São João da Barra	Norte Fluminense	−41.071213	−21.669826
EU38	RBR 57968	São Francisco de Itabapoana	Norte Fluminense	−41.101203	−21.582566
EU39	RBR 57969	São Francisco de Itabapoana	Norte Fluminense	−41.062051	−21.567322
EU40	RBR 57970	São Francisco de Itabapoana	Norte Fluminense	−40.983651	−21.390232
EU41	RBR 57971	São Francisco de Itabapoana	Norte Fluminense	−40.964658	−21.314880
EU42	RBR 57972	São Francisco de Itabapoana	Norte Fluminense	−40.965088	−21.324449

**FIGURE 1:**
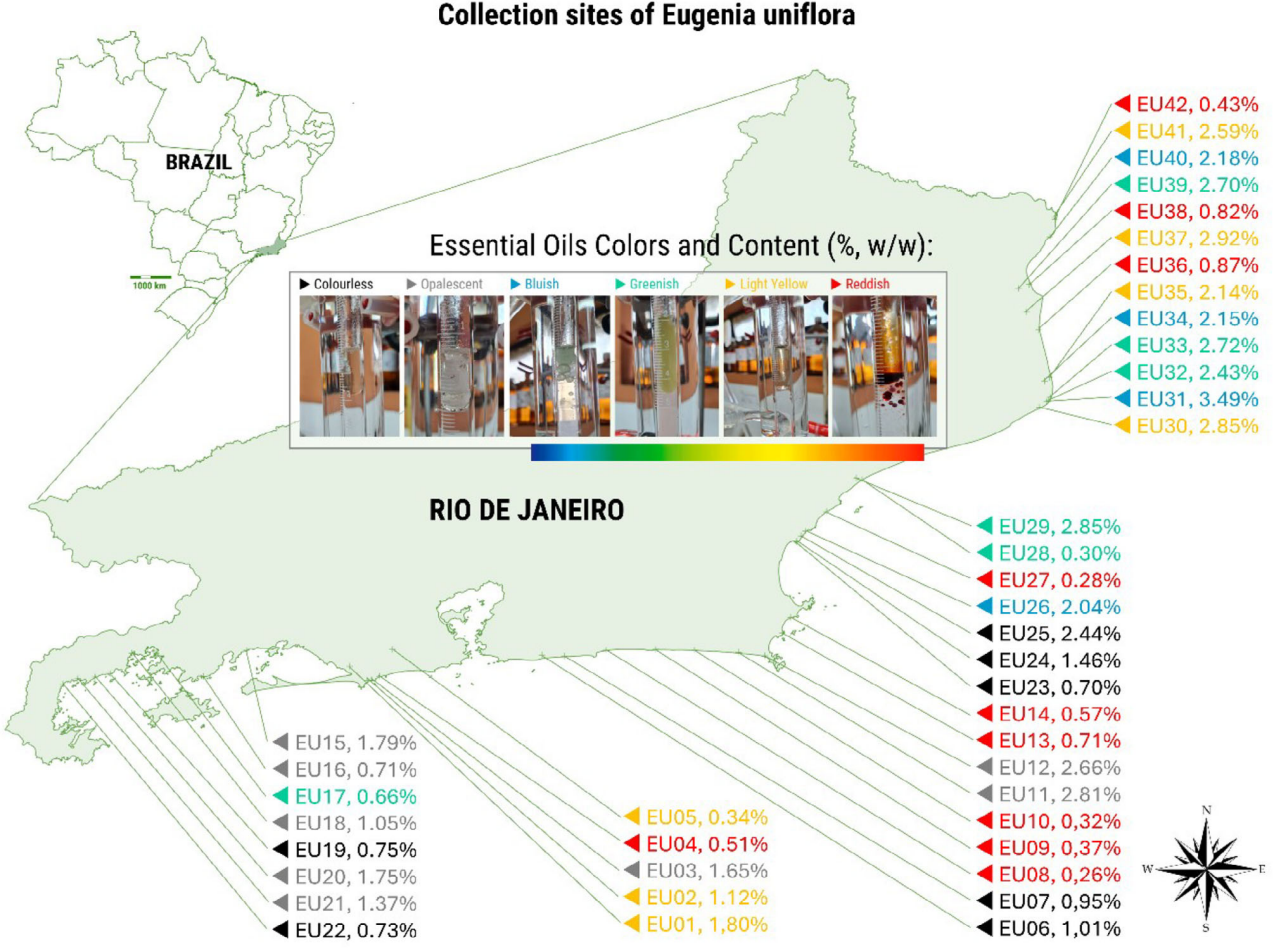
Collection sites of 42 *Eugenia uniflora* plants from the coastal region of Rio de Janeiro, Brazil. Sample codes (EU01–EU42) refer to collection IDs listed in Table 1, followed by essential oil content (% w/w). Oil color classifications are illustrated in the central panel and correspond to the visual appearance of the oils obtained from each sample.

Reddish‐colored oils were predominantly associated with low yields, as observed in EU08 (0.26%) and EU10 (0.32%). In contrast, samples with bluish and greenish tones, such as EU31 (3.49%) and EU40 (2.18%), exhibited relatively high essential oil contents (Figure 1; Table S1).

**FIGURE 2:**
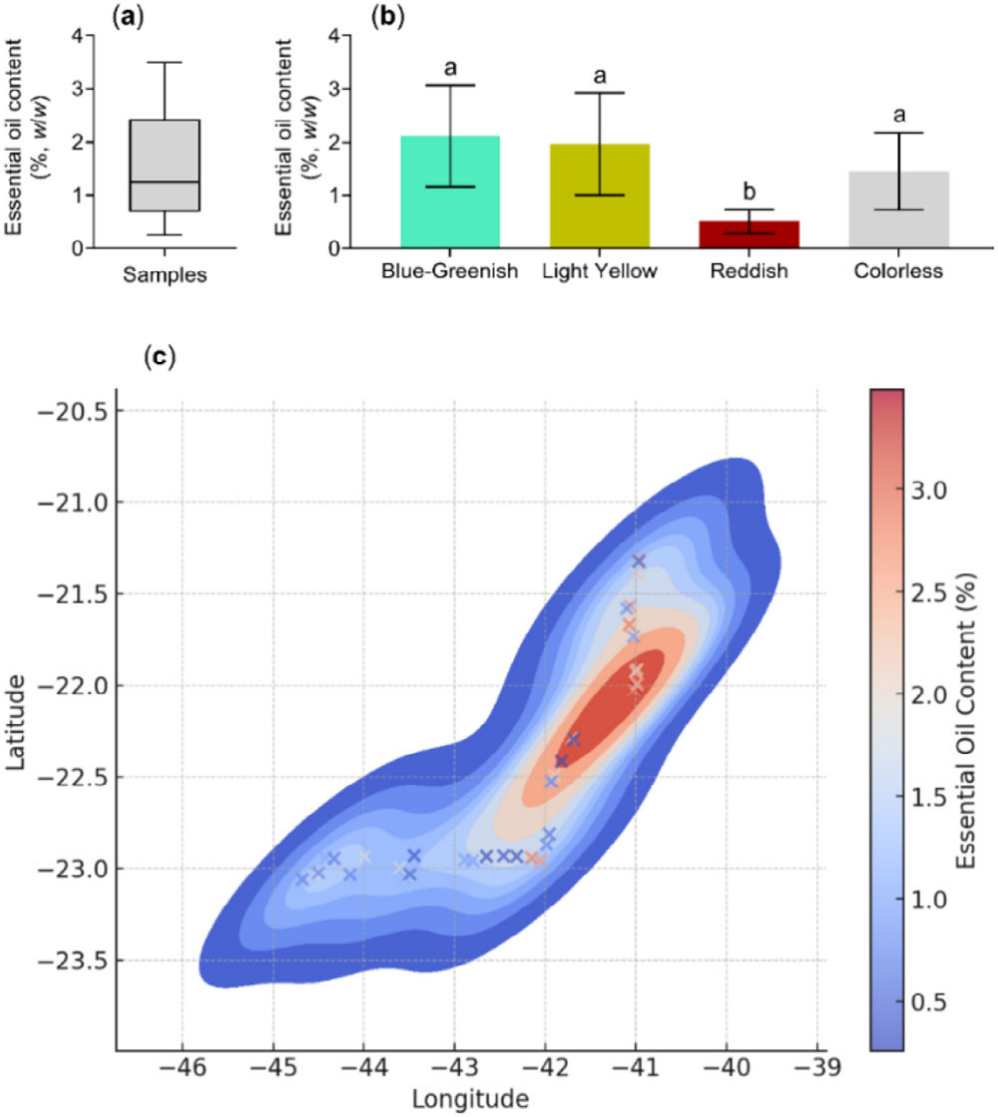
Statistical descriptions of essential oil content (% w/w) in *Eugenia uniflora* samples from the coastal region of Rio de Janeiro: (a) distribution of oil content; (b) content (%) in relation to oil color; and (c) heatmap based on geographic coordinates and essential oil content. Boxplots show quartiles; the central line represents the median. Different letters indicate statistically significant differences based on Tukey's test at 5% probability.

The descriptive analysis based on the essential oil content of the different samples showed a median of 1.2%, with 50% of the samples distributed between 0.9% and 2.4% (Figure 2a). Statistical analysis of essential oil content in relation to oil color revealed that reddish samples exhibited significantly lower mean values (0.3%) compared to the other colors (Figure 2b). The heatmap displayed the spatial distribution of essential oil content (%) and revealed a significant concentration of higher yields between latitudes −22.5 and −21.5 and longitudes −42 and −40, encompassing parts of the Baixadas Litorâneas and the Norte Fluminense Regions (Figure 2c).

Chromatographic analysis allowed the construction of a data matrix with a total of 72 substances detected and integrated, of which 49 were identified, across 42 essential oil samples obtained from *E. uniflora* leaves (Table S2). Among the 72 substances, 30 were selected for the study based on their frequency (> 25%) and/or concentration (> 5%) (Table 2).

**TABLE 2:** Descriptive statistics of the chemical composition of essential oils from *Eugenia uniflora* leaves, based on GC–FID and GC–MS analyses.

Compounds^a^	Class	Min	Max	Mean	±SD	*f* (%)
(*Z*)‐β‐Ocimene	MH	0.6	5.7	2.3	1.9	17
(*E*)‐β‐Ocimene	MH	0.3	12.7	2.6	3.5	40
*trans*‐Linalool oxide acetate	OM	5.9	5.9	5.9	—	2
β‐Elemene	SH	0.7	8.6	4.2	2.3	81
(*E*)‐Caryophyllene	SH	0.6	13.1	3.4	3.0	86
γ‐Elemene	SH	0.5	5.3	1.7	1.1	74
*allo*‐Aromadendrene	SH	0.4	16.7	7.3	5.9	38
Germacrene D	SH	0.9	5.5	2.2	1.2	74
Viridiflorene	SH	0.4	3.0	1.3	0.7	26
Curzerene	OS	1.6	80.8	43.3	24.4	43
Bicyclogermacrene	SH	0.7	16.3	3.9	3.5	52
Germacrene A	SH	0.3	7.6	1.9	3.2	12
δ‐Cadinene	SH	1.7	10.7	4.8	2.8	24
Germacrene B	SH	3.5	19.5	6.9	3.3	76
Spathulenol	OS	0.4	40.5	8.8	11.6	86
Caryophyllene oxide	OS	0.5	11.4	4.8	3.5	31
Viridiflorol	OS	0.4	3.8	1.1	0.9	45
Muurola‐4,10(14)‐dien‐1‐β‐ol	OS	1.2	10.1	4.5	2.8	21
Selina‐1,3,7(11)‐trien‐8‐one	OS	0.7	58.8	30.1	19.2	60
*allo*‐Aromadendrene epoxide	OS	0.5	3.7	1.5	0.9	29
*epi*‐α‐Muurolol	OS	0.7	5.6	3.2	1.6	21
α‐Cadinol	OS	0.8	14.0	4.2	3.9	31
Atractylone	OS	0.7	3.6	2.3	0.7	29
Germacrone	OS	0.3	11.8	2.9	3.5	40
Amorpha‐4,9‐dien‐2‐ol	OS	0.3	38.3	5.3	11.7	24
Selina‐1,3,7(11)‐trien‐8‐one epoxide	OS	1.4	45.8	26.4	14.3	55
NI		3.6	9.8	5.6	2.4	24
NI		1.2	14.3	8.0	5.1	21
NI		0.9	5.4	2.7	2.4	7
NI		0.7	10.2	5.0	3.1	19

Abbreviations: *f*, frequency of occurrence in which the compound was detected; mean, arithmetic mean; MH, monoterpene hydrocarbons; Min, minimum value detected under quantification conditions; NI, no identified; OM, oxygenated monoterpenes; OS, oxygenated sesquiterpenes; SD, standard deviation; SH, sesquiterpene hydrocarbons.

^a^Selection of compounds with frequency > 25% and/or concentration > 5% (based on flame ionization detector, FID).

**FIGURE 3:**
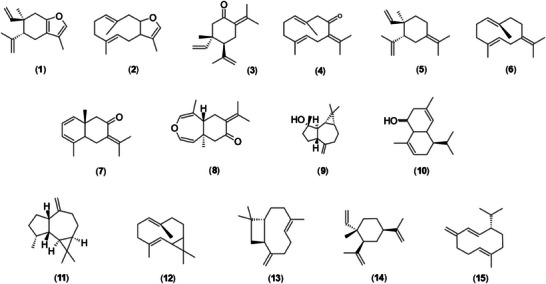
Chemical structures of compounds commonly found in the *Eugenia uniflora* essential oil.

The essential oils analyzed exhibited a terpene‐rich chemical profile, such as curzerene (**1**), which ranged from 1.6% to 80.8% in concentration and was present in 43% of the samples. Other prominent compounds included selina‐1,3,7(11)‐trien‐8‐one (**7**) and its epoxidized form (**8**), with concentrations ranging from 0.7% to 58.8% and 1.4% to 45.8%, and frequencies of 60% and 55%, respectively. Additionally, the oxygenated sesquiterpene spathulenol (**9**) exhibited concentrations between 0.4% and 40.5%, occurring in 86% of the samples. The compounds (*E*)‐caryophyllene (**13**) and β‐elemene (**14**), with frequencies of 86% and 81%, respectively, as well as germacrene B (**6**) with a frequency of 76%, and *γ*‐elemene (**5**) and germacrene D (**15**), both at 74%, showed broad distribution among the analyzed samples, though in intermediate proportions (Table 2; Figure 3).

**FIGURE 4:**
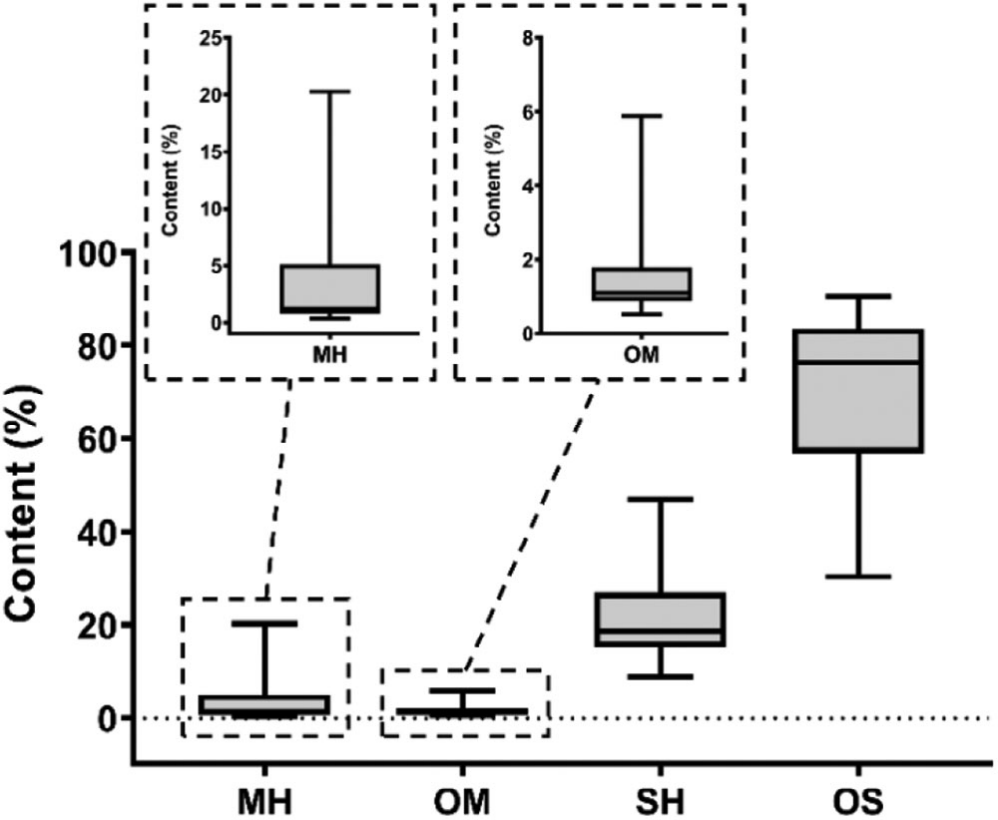
Boxplot representation of the proportions of monoterpene hydrocarbons (MH), oxygenated monoterpenes (OM), sesquiterpene hydrocarbons (SH), and oxygenated sesquiterpenes (OS) in essential oil samples from *Eugenia uniflora* collected in situ. Insets highlight the distribution of MH and OM due to their lower concentration ranges. Boxes represent the interquartile range, the horizontal line indicates the median, and whiskers extend to the minimum and maximum values.

Oxygenated sesquiterpenes exhibited the highest mean concentration (69%), predominating in the composition of the essential oils, followed by sesquiterpene hydrocarbons with a mean concentration of 21%. Oxygenated monoterpenes (MO) and monoterpene hydrocarbons (MH) were present in lower proportions (Figure 4).

**FIGURE 5:**
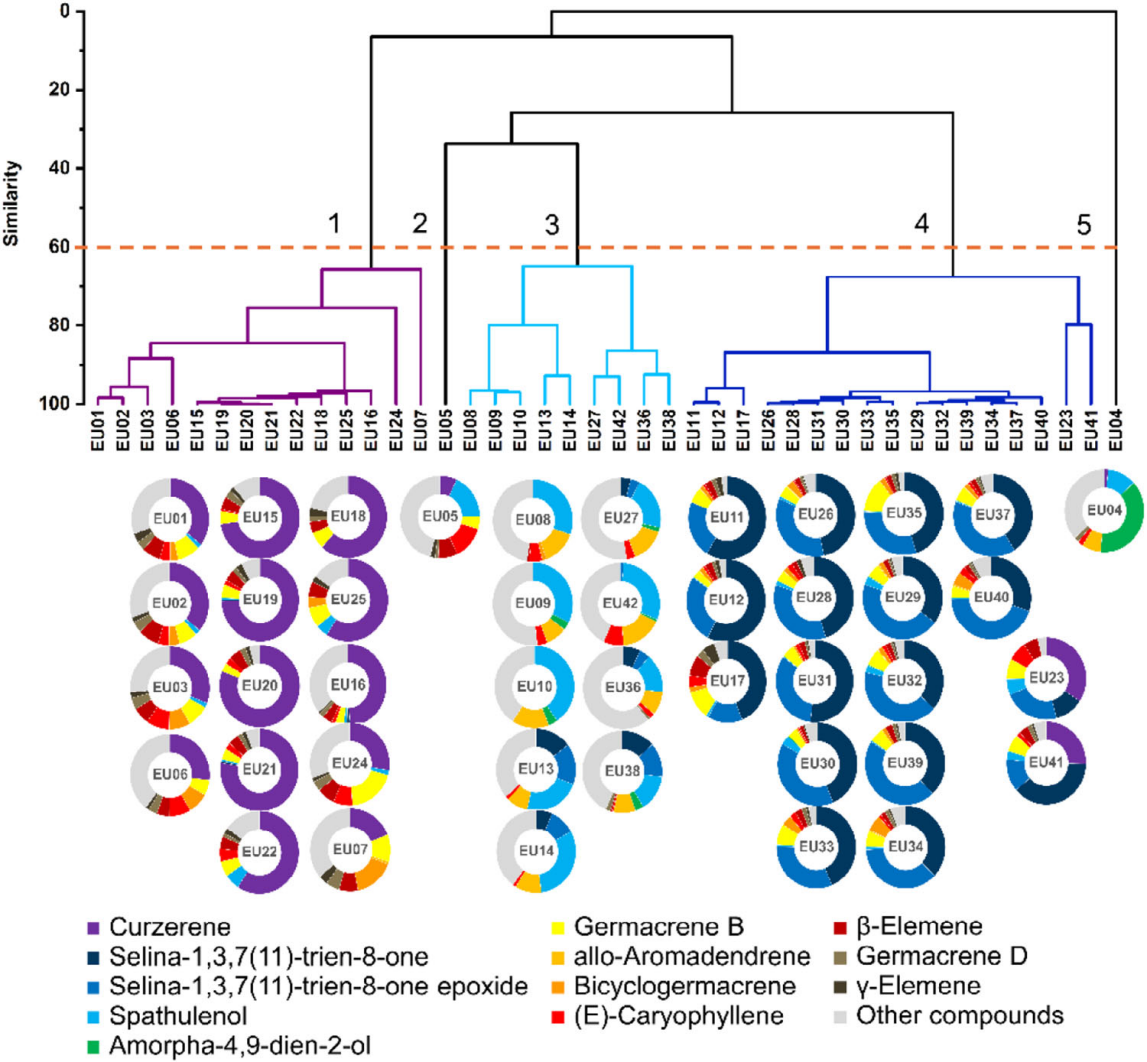
Colored donut charts representing the essential oil profiles of 42 *Eugenia uniflora* samples collected from different locations in the state of Rio de Janeiro, based on 12 major compounds. The dendrogram (a) shows hierarchical cluster analysis using the UPGMA method and Pearson correlation coefficient between independent variables (samples) and dependent variables (relative abundances of 30 compounds). Clusters were defined based on a 60% similarity cut‐off (dashed red line). Donut chart colors indicate the relative proportion of each compound (chemoarrays).

The data matrix constructed from the samples and chemical composition of the essential oils was subjected to hierarchical clustering analysis (Figure 5). Five groups were formed based on the similarity of the essential oil chemical profiles. The calculated cophenetic correlation was 0.92, and the cut‐off line was arbitrarily set at 60% similarity. Analysis of the doughnut charts in association with the dendrogram revealed three major chemotype profiles: Group 1, rich in curzerene (**1**); Group 3, rich in spathulenol (**9**) and *allo*‐aromadendrene (**11**); and Group 4, rich in selina‐1,3,7(11)‐trien‐8‐one (**7**) and its epoxidized form (**8**). Sample EU05, primarily composed of spathulenol (**9**) and (*E*)‐caryophyllene (**13**), and Sample EU04, mainly containing amorpha‐4,9‐dien‐2‐ol (**10**), constituted isolated groups, identified as Groups 2 and 5, respectively (Figure 5).

**FIGURE 6:**
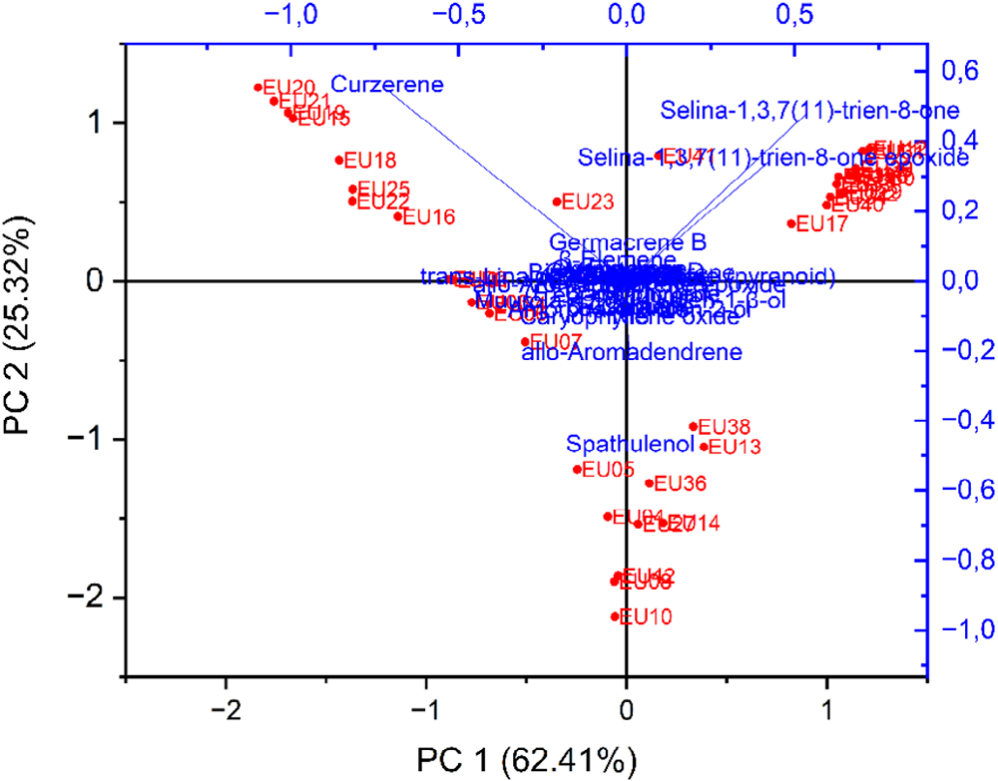
Principal component analysis (PCA) based on the essential oils of 42 *Eugenia uniflora* samples collected from different locations in the state of Rio de Janeiro (scores in red) and relative abundances of 30 compounds from essential oils (loadings in blue), jointly displayed in the biplot.

The biplot from the principal component analysis (PCA) accounted for more than 87% of the total variance across the two components considered: PC1 with 62.41% and PC2 with 25.32% of the variance. It was possible to observe the contribution of curzerene (**1**), selina‐1,3,7(11)‐trien‐8‐one (**7**), and spathulenol (**9**) to the dispersion of the samples and the concomitant formation of clusters, corroborating the results of the hierarchical clustering analysis (Figure 6).

**FIGURE 7:**
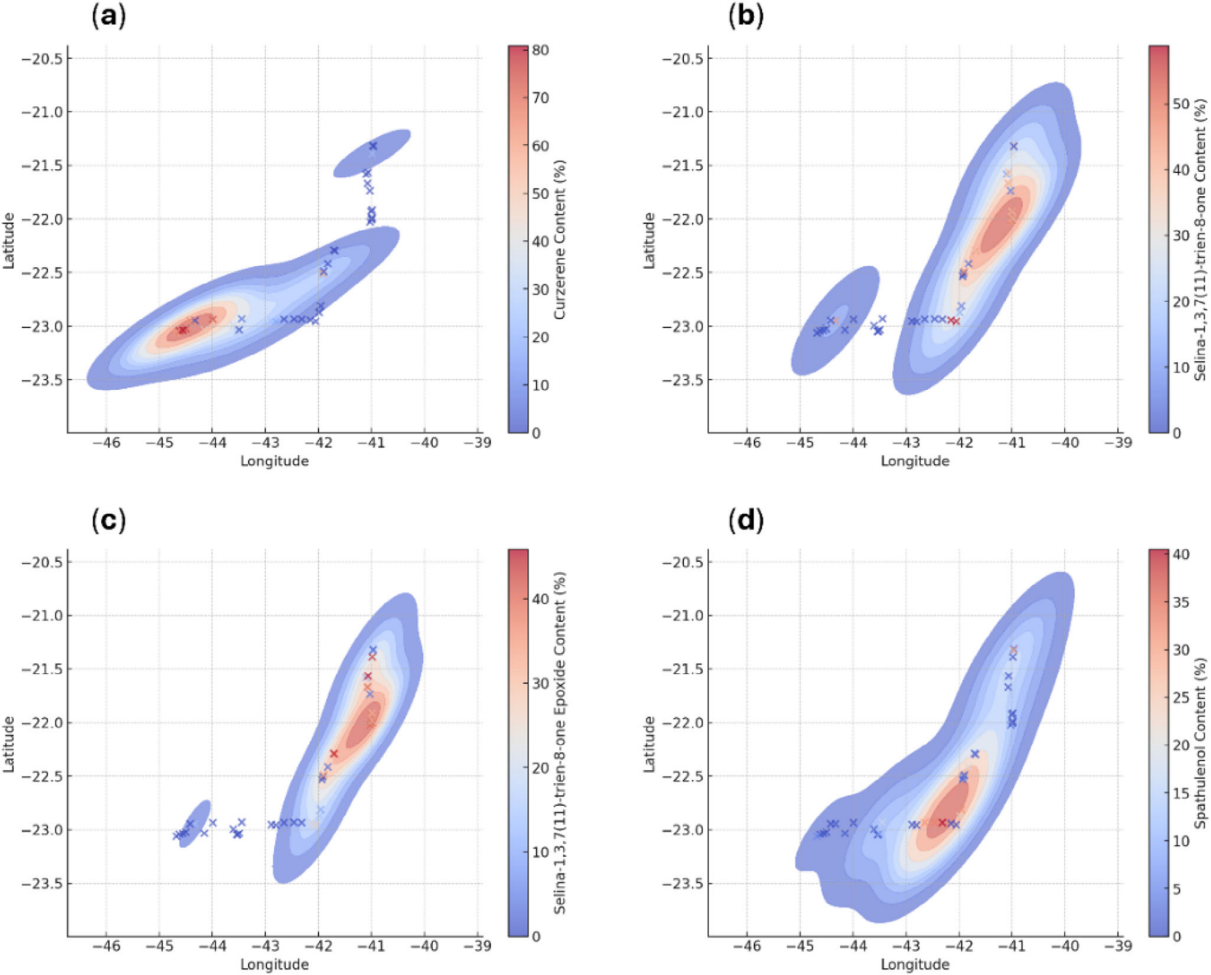
Heatmaps showing the spatial distribution of compound proportions (% w/w) across the collection sites of *Eugenia uniflora*. Red tones indicate higher concentrations, and blue tones represent lower values. Data points (×) correspond to the geographic coordinates where plants were collected and essential oil samples were obtained. Each panel represents the distribution of: (a) curzerene, (b) selina‐1,3,7(11)‐trien‐8‐one, (c) selina‐1,3,7(11)‐trien‐8‐one epoxide, and (d) spathulenol.

Curzerene exhibited a specific distribution pattern, with higher proportions restricted to the Costa Verde region, in the southern coastal area of the state of Rio de Janeiro, specifically in the municipalities of Paraty, Angra dos Reis, and Mangaratiba (Figure 7a). Selina‐1,3,7(11)‐trien‐8‐one (**7**) and its epoxidized (**8**) form showed higher proportions in the Baixadas Litorâneas and Norte Fluminense regions, particularly in the municipalities of Rio das Ostras and Macaé (Figure 7b,d). Spathulenol (**9**), in turn, showed the highest proportions in the Metropolitana and Baixadas Litorâneas regions, encompassing the municipalities of Rio de Janeiro, Maricá, Saquarema, Araruama, Arraial do Cabo, Cabo Frio, Armação dos Búzios, and Rio das Ostras (Figure 7d).

Table 3 presents the classification of *E. uniflora* samples into distinct chemotypes based on the dominance of one, two, or three major chemical constituents. The predominant chemotypes were (i) curzerene and (v) selina‐1,3,7(11)‐trien‐8‐one/selina‐1,3,7(11)‐trien‐8‐one epoxide, both identified in 12 samples. Other chemotypes observed included (ii) selina‐1,3,7(11)‐trien‐8‐one in three samples, (iii) spathulenol in four samples, and the chemotypes (iv) amorpha‐4,9‐dien‐2‐ol, (vi) curzerene/germacrene B, (vii) curzerene/selina‐1,3,7(11)‐trien‐8‐one epoxide, (viii) spathulenol/*allo*‐aromadendrene, and (ix) selina‐1,3,7(11)‐trien‐8‐one/curzerene/selina‐1,3,7(11)‐trien‐8‐one epoxide, each found in one sample (Table 3). Some samples exhibited undefined or mixed chemical profiles, with no clear dominance of a single major compound.

**TABLE 3:** Characterization of chemotypes based on the dominance of major compounds present in the essential oils of *Eugenia uniflora* from in situ collected plants.

No.	Chemotype	Type^a^	Plant ID (% compound)^b^
(i)	Curzerene	A	EU01(35), EU02 (36), EU03 (31), EU06 (27), EU15 (73), EU16 (49), EU18 (62), EU19 (74), EU20 (81), EU21(78), EU22 (59), EU25 (59).
(ii)	Selina‐1,3,7(11)‐trien‐8‐one	A	EU11 (59), EU12 (58), EU17 (44).
(iii)	Spathulenol	A	EU08 (31), EU09 (32), EU10 (41), EU14 (31)
(iv)	Amorpha‐4,9‐dien‐2‐ol	A	EU04 (38)
(v)	Selina‐1,3,7(11)‐trien‐8‐one/Selina‐1,3,7(11)‐trien‐8‐one epoxide	A/B	EU26 (47/35), EU28 (45/35), EU29 (35/45), EU30 (44/39), EU31 (52/33), EU32 (36/42), EU33 (44/32), EU34 (37/37), EU35 (45/31), EU37 (41/40), EU39 (38/46), EU40 (30/44)
(vi)	Curzerene/ Germacrene B	A/B	EU24 (27/20)
(vii)	Curzerene/ Selina‐1,3,7(11)‐trien‐8‐one epoxide	A/B	EU23 (34/23)
(viii)	Spathulenol/*allo*‐aromadendrene	A/B	EU42 (30/ 17)
(ix)	Selina‐1,3,7(11)‐trien‐8‐one/ Curzerene/ Selina‐1,3,7(11)‐trien‐8‐one epoxide	A/B/C	EU41 (38/25/13)
	Undefined or mixed	M	EU05, EU07, EU13, EU27, EU36, EU38

*Note*: Values in parentheses represent the relative concentration (%) of the indicated compound(s).

Abbreviation: No., chemotype number.

^a^Type—number of dominant major compounds.

^b^ID—plant identification code corresponding to Table S2.

## 3 Discussion

The *E. uniflora* plants in this study were distributed across locations with distinct landscape and phytophysiognomic conditions, although all collections were conducted in coastal regions of the state of Rio de Janeiro (Figure S1). The Norte Fluminense region is characterized by *restinga* formations, nutrient‐poor sandy soils, and shrub vegetation adapted to water stress [23], whereas the Costa Verde region features more rugged terrain, with steep slopes and deep valleys, and supports dense, humid vegetation favored by higher rainfall levels and lower influence of sandy soils [24].

Previous studies have highlighted that edaphoclimatic factors such as temperature, solar radiation, and water availability are key determinants in the biosynthesis of specialized metabolites [3]. The interaction between genotype and environment clearly leads to phenotypic variation within the species, for example, in terms of essential oil content (%) and chemical profile. In addition, *E. uniflora* exhibits a mixed reproductive system, with both allogamy and autogamy, the former being strongly influenced by pollinators, which in turn promotes greater genetic segregation and, consequently, increased variability among offspring [25].

This study revealed significant variation in essential oil content (Figures 1 and 2a), likely in response to genotype–environment interaction. In addition, the results showed that oil contents above 2% were predominantly obtained from plants sampled along the northern coast (Norte Fluminense region), whereas values below 2% were mostly associated with plants collected from the southern coast, specifically the Costa Verde region (Figures 1 and 2c). This finding provides strong evidence that genotype–environment interaction contributes to the variation in essential oil content (%) observed in *E. uniflora* leaves. Previous studies have reported contrasting essential oil yields from *E. uniflora* leaves; for example, Victoria et al. [26] reported yields as low as 0.10%, while Pinheiro et al. [27] reported 2.1%.

Similarly, the color of the essential oils, which is indicative of variation in chemical composition, also varied considerably—except in the Costa Verde region, where colorless and opalescent essential oils predominated (Figure 1). An interesting aspect was the association between reddish coloration and lower essential oil content, a phenomenon previously reported by Chang et al. [28]. These authors attributed this hue to the presence of oxidizable compounds such as furanodienes, which are subject to thermal and oxidative rearrangements. In the present study, we observed that reddish‐colored essential oils were all grouped within cluster 3 (Figure 5), characterized by high concentrations of spathulenol (**9**) and *allo*‐aromadendrene (**11**).

Before proceeding with a more detailed discussion of essential oil profiles, it is important to highlight the study by Santos et al. [21], which demonstrated that cyclic sesquiterpenes of the “1,5‐diene type” can undergo *Cope rearrangement* during chromatographic separation at high temperatures. Under such conditions, cyclic sesquiterpenes such as furanodiene (**2**), germacrone (**4**), and germacrene B (**6**), present in the essential oils of *E. uniflora*, undergo rearrangement to form curzerene (**1**), β‐elemene (**3**), and *γ*‐elemene (**5**), respectively [29].

Sesquiterpenes were the main constituents of the essential oil of *E. uniflora*, with curzerene being the most abundant compound, exhibiting concentrations ranging from 1.6% to 80.8%. Essential oils of *E. uniflora* rich in curzerene have already been reported in the literature [18, 28]. Other major compounds included selina‐1,3,7(11)‐trien‐8‐one (**7**) and its epoxidized (**8**) form, with concentrations ranging from 0.7% to 58.8% and occurring at high frequency among the samples. This dominance pattern has also been described by Ascari et al. [30], who identified similar chemotypes in *E. uniflora* populations from southern Brazil.

Biodiversity represents a universal heritage of immeasurable value, particularly considering its numerous potential applications that remain unexplored. Even the limited studies conducted thus far reveal promising prospects. For instance, furanodiene exhibits anticancer activity through the induction of apoptosis and modulation of signaling pathways such as MAPKs and NF‐κB [31]. Spathulenol has demonstrated anti‐inflammatory, immunomodulatory, antinociceptive, and antimycobacterial effects [32, 33, 34]. Moreover, selina‐1,3,7(11)‐trien‐8‐one and its epoxide derivative have shown selective cytotoxicity against human tumor cell lines, as well as antimicrobial activity against Gram‐positive bacteria and *Candida albicans* [30, 35].

The predominance of oxygenated sesquiterpenes in the chemical composition (mean of 69%) reinforces the chemical profile previously described for the species [17, 20, 27, 30]. Recent studies highlight that the oxygenation of sesquiterpenes may be associated with ecological adaptations of the plant, influencing its biological activity [36, 37].

In this study, it was also possible to describe, through heatmaps (Figure 7), that the curzerene (**1**) and selina‐1,3,7(11)‐trien‐8‐one (**7**) were distributed across geographically distinct locations. These compounds were found in higher proportions in samples obtained from plants in the Costa Verde and the Baixadas Litorâneas/Norte Fluminense regions, respectively. Although the effect of genotype–environment interaction on the diversity of chemical profiles in *E. uniflora* was not a working hypothesis in this study, there is evidence in the literature that plants respond chemically to the presence of insects [38, 39, 40] and/or to specific edaphoclimatic conditions [41, 42, 43].

This study demonstrated that the essential oils exhibited chemical diversity, as shown by the hierarchical clustering analysis, which revealed five distinct chemical profiles (i.e., five groups), three of which comprised the majority of the samples (Figure 5). These results were supported by the PCA (Figure 6). Curzerene (Group 1), spathulenol and *allo*‐aromadendrene (Group 3), and selina‐1,3,7(11)‐trien‐8‐one and its epoxide (Group 4) were the most relevant compounds contributing to group formation and to the classification of the two most representative chemotypes out of a total of nine observed, namely, the (i) curzerene and (v) selina‐1,3,7(11)‐trien‐8‐one/selina‐1,3,7(11)‐trien‐8‐one epoxide chemotypes, both identified in 12 samples (Table 3). This pattern of chemical distribution is consistent with the findings of Gonçalves et al. [22], who highlighted the existence of at least three major chemotypes for the species.

The information presented in this study confirms the importance of genetic background in explaining this diversity. Otherwise, how could one explain two plants growing under the same landscape conditions—only 1.1 km apart—exhibiting markedly different essential oil contents, colors, and chemical profiles, as observed in samples EU41 (2.59%, light yellow, Group 4, chemotype: selina‐1,3,7(11)‐trien‐8‐one/curzerene/selina‐1,3,7(11)‐trien‐8‐one epoxide) and EU42 (0.43%, reddish, Group 3, spathulenol/*allo*‐aromadendrene)? The explanation is evidently grounded in the genetic value represented by each specimen, which reinforces the chemical diversity of the population studied in situ.

## 4 Conclusion

Overall, the results indicate clear diversity in the type, color, and chemical composition of *E. uniflora* essential oils found along the coastal regions of the state of Rio de Janeiro. The evidence points to a possible geographical influence—considering the distinct phytophysiognomies and landscapes—and the existence of different plant populations affecting the yield and chemical profiles of the essential oil samples analyzed. These findings highlight the need for further research on both in situ populations and ex situ collections of *E. uniflora*, including DNA analysis, to better understand their impact on essential oil production and composition.

## 5 Experimental Section

### 5.1 Research Authorization

This study was registered in the National System for the Management of Genetic Heritage and Associated Traditional Knowledge (SisGen, registration codes AAA4A76 and A40DB1C) and obtained the necessary authorizations for field material collection from the State Institute for the Environment of Rio de Janeiro (INEA, authorization 023/2021) and the Chico Mendes Institute for Biodiversity Conservation (ICMBio, authorization code 78469‐1).

### 5.2 Prospecting and Plant Collection

The collection area was initially selected based on a preliminary analysis of the terrain and vegetation, conducted using the Google Earth software. The sampling method consisted of the random selection of *E. uniflora* L. (Myrtaceae) individuals within a previously defined area, ensuring a minimum distance of 200 m between native specimens. The in situ collections were carried out in the state of Rio de Janeiro (Brazil). The first collection included 22 plants sampled between the municipalities of Paraty and Armação de Búzios, from September 3 to November 7, 2021, and the second included 20 individuals collected between the municipalities of Rio das Ostras and São Francisco de Itabapoana, from September 23 to November 18, 2022 (Table 1; Figure 1).

The collected material (branches containing leaves, inflorescences, and fruits) was in either vegetative or reproductive stage and was identified using the code “EU” followed by a sequential number from 1 to 42, corresponding to each plant sampled in the field. All specimens were georeferenced, and their information is presented in Table 1. Branches containing flowers and/or fruits were sent to the herbarium of UFRRJ for preparation of voucher specimens, registration, and assignment of respective accession numbers (Table 1). The leaves intended for essential oil extraction were subjected to a drying process and stored according to the literature [4], until the time of essential oil extraction.

### 5.3 Distillation of Essential Oils

Essential oil extraction was carried out at the Laboratory of Aromatic and Medicinal Plants of the Federal Rural University of Rio de Janeiro (UFRRJ), located in the municipality of Seropédica. The essential oils were obtained by hydrodistillation using a Clevenger‐type apparatus. For each extraction, 30 g of previously ground dried leaves were placed in a 1 L round‐bottom flask containing 400 mL of distilled water. The distillation process was conducted for 2 h and 30 min, with a steam flow rate of 3 mL/min. The complete separation and recovery of the essential oil, as well as the quantification of yield (%, w/w) based on the dry weight of the leaves, were performed as described in the literature [4].

### 5.4 GC–FID and GC–MS Analyses

The samples were prepared by diluting the essential oils in absolute ethanol at a concentration of 10 mg/mL. Subsequently, 1 µL of each sample was injected into a gas chromatograph (5890 Series II, Hewlett‐Packard, USA) equipped with a flame ionization detector (GC–FID), operating in split mode (1:20). Compound separation was performed using a fused silica capillary column with a stationary phase composed of 5% phenyl and 95% dimethylpolysiloxane (30 m × 0.25 mm × 0.25 µm i.d.). The carrier gas flow rate and the temperature program for the column, injector, and detector followed the conditions previously described [4]. The same sample and volume (1 µL) were injected into a gas chromatograph coupled to a mass spectrometer (GC–MS), model QP‐2010 Plus (Shimadzu, Japan). The temperature program for the column and the injector and interface temperatures were identical to those used in the GC–FID analysis, as previously described [4]. The mass spectrum was acquired using a quadrupole detector, operating at 70 eV, with a scan range of 40–4100 *m*/*z* and a scan rate of 0.5 scans/s. Quantification of the compounds present in the essential oil was based on the peak areas obtained by GC–FID, with values converted into relative percentages using GC–MS Solution software v.2.53 (Shimadzu). Compound identification was performed by GC–MS through the determination of the linear retention index (LRI), calculated from a homologous series of *n*‐alkanes (C7–C30) injected under the same analytical conditions as the samples [44], and by comparison of the mass spectra with the NIST database (2023) and literature [45].

### 5.5 Essential Oil Data Matrix

Based on the samples collected along the coastal region of the state of Rio de Janeiro, a data matrix was constructed containing 42 essential oils (independent variables) and 49 compounds identified in the analyzed samples (dependent variables). To highlight the variables contributing most to the total data variability, the following criteria were applied: only substances present at proportions equal to or greater than 5% in at least one of the essential oils and/or with a frequency (*f* %) greater than 25% were considered.

### 5.6 Chemotype Characterization

To standardize the nomenclature of chemotypes (CT), the classification key previously proposed by de Medeiros et al. [4] was used. This system is based on the dominance of major compounds in each volatile oil sample, considering one (A), two (A/B), or three (A/B/C) dominant substances in relation to the other constituents.

### 5.7 Statistical Analyses

*Descriptive and inferential statistics*: The minimum, maximum, mean values, and the relative frequency of chemical compounds in the essential oils were organized and presented using column and boxplot charts. To facilitate visual analysis and chemometric representation, doughnut charts were constructed following the approach described by de Medeiros et al. [4] and Singh et al. [46], considering the 12 most abundant compounds in the data matrix. One‐way analysis of variance (ANOVA) and Tukey's post hoc test (5%) were applied to color and essential oil yield (%) data. The statistical analyses and construction of tables and graphs were carried out using Microsoft Excel and GraphPad Prism 9. Geospatial analyses were performed using Python 3.9, with support from the NumPy [47], Pandas [48], Matplotlib [49], Seaborn [50], and SciPy [51] libraries. Heatmaps were generated using the Seaborn *kdeplot* method, and spatial interpolation was conducted using *griddata* from SciPy. Part of the data analysis and figure generation was supported by the artificial intelligence tool ChatGPT (OpenAI, San Francisco, CA, USA), particularly in the development and refinement of Python scripts used for spatial visualization and interpolation [52].

*Multivariate analysis*: The data matrices were subjected to UPGMA clustering using Pearson correlation as the distance metric for dendrogram construction. The quality of the fit between phenetic and cophenetic matrices was assessed using the cophenetic correlation coefficient, as described by Sokal and Rohlf [53], and the optimal number of groups was defined by considering a minimum similarity of 60% among samples. In addition, a PCA was performed, and a biplot was constructed to represent the scores and factor loadings of the analyzed variables. Hierarchical clustering and PCA were performed using Origin software v.2022b (OriginLab Corporation, Northampton, MA, USA).

## Conflicts of Interest

The authors declare no conflicts of interest.

## Supporting information




**Supporting File 1**: cbdv70249‐sup‐0002‐TableS2.xlsx

**Supporting File 2**: cbdv70249‐sup‐0001‐SuppMat.docx

## Data Availability

The data that support the findings of this study are available from the corresponding author upon reasonable request.
