# Supplementary material for: Chemodiversity of Essential Oils From Eugenia uniflora L. Collected in Different Phytophysiognomies of the Coastal Region of Rio de Janeiro
Source: Chem Biodivers. 2025 Jul 17;22(11):e01390. doi: 10.1002/cbdv.202501390 (PMC12629159; doi:10.1002/cbdv.202501390)
Supplement: Supplementary file 2 — Supporting File 2: cbdv70249‐sup‐0001‐SuppMat.docx [file CBDV-22-e01390-s002.docx]

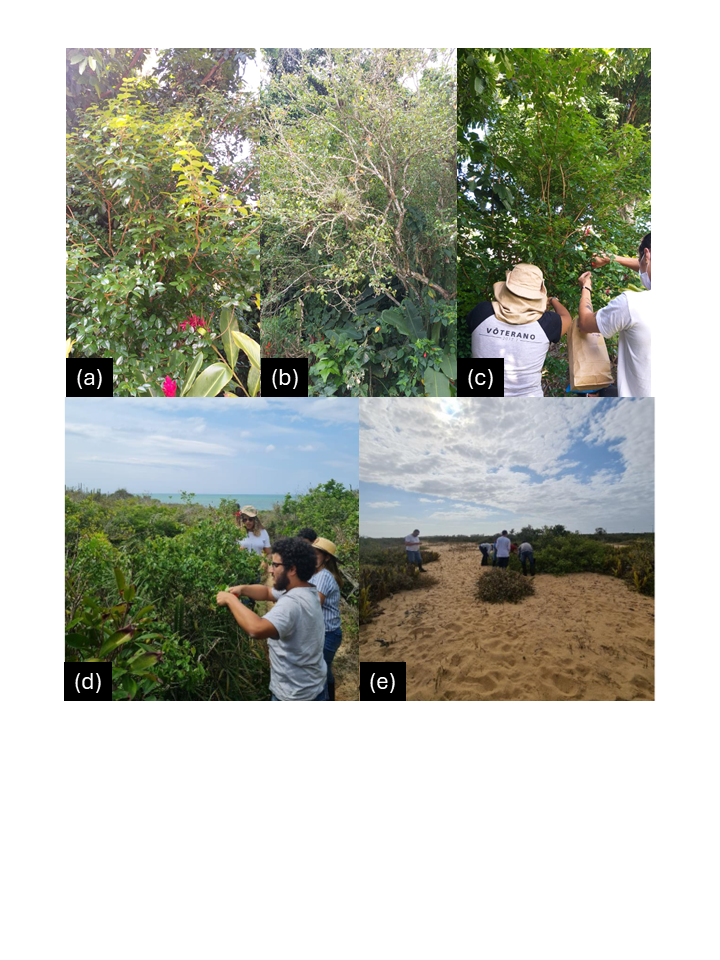


**Figure S1.** Landscape and phytophysiognomy of the Costa Verde (a–c) and the Baixadas Litorâneas/Norte Fluminense regions (d–e) in the state of Rio de Janeiro, Brazil. Panels (a) and (b) show the tree-like growth habit and vertical structure of E. uniflora plants. Panel (c) shows the sampling moment. Panels (d) and (e) highlight the shrubby and creeping growth habits of E. uniflora in sandy soil environments.

**Table S1.** Essential oil content (%, w/w) and color of oils obtained from *E. uniflora* leaves collected in different locations across the state of Rio de Janeiro.

| Plant ID | Essential oil content (w/w, %) | Essential Oil Color |
| --- | --- | --- |
| EU01 | 1.80 | Light Yellow |
| EU02 | 1.12 | Light Yellow |
| EU03 | 1.65 | Opalescent |
| EU04 | 0.51 | Reddish |
| EU05 | 0.34 | Light Yellow |
| EU06 | 1.01 | Colorless |
| EU07 | 0.95 | Colorless |
| EU08 | 0.26 | Reddish |
| EU09 | 0.37 | Reddish |
| EU10 | 0.32 | Reddish |
| EU11 | 2.81 | Opalescent |
| EU12 | 2.66 | Opalescent |
| EU13 | 0.71 | Reddish |
| EU14 | 0.57 | Reddish |
| EU15 | 1.79 | Opalescent |
| EU16 | 0.71 | Opalescent |
| EU17 | 0.66 | Greenish |
| EU18 | 1.05 | Opalescent |
| EU19 | 0.75 | Colorless |
| EU20 | 1.75 | Opalescent |
| EU21 | 1.37 | Opalescent |
| EU22 | 0.73 | Colorless |
| EU23 | 0.70 | Colorless |
| EU24 | 1.46 | Colorless |
| EU25 | 2.44 | Colorless |
| EU26 | 2.04 | Bluish |
| EU27 | 0.28 | Reddish |
| EU28 | 0.30 | Greenish |
| EU29 | 2.51 | Greenish |
| EU30 | 2.85 | Light Yellow |
| EU31 | 3.49 | Bluish |
| EU32 | 2.43 | Greenish |
| EU33 | 2.72 | Greenish |
| EU34 | 2.15 | Bluish |
| EU35 | 2.14 | Light Yellow |
| EU36 | 0.87 | Reddish |
| EU37 | 2.92 | Light Yellow |
| EU38 | 0.82 | Reddish |
| EU39 | 2.70 | Greenish |
| EU40 | 2.18 | Bluish |
| EU41 | 2.59 | Light Yellow |
| EU42 | 0.43 | Reddish |
